# Ingrowing Toe Nail

**Published:** 1874-03

**Authors:** J. Q. A. Hudson


Vol. IV.—MARCH, 1874.—No. 3.

### Original Communications.

### INGROWING TOE NAIL.

BY J. Q. A. HUDSON, M.D.

*Read before the Meigs & Mason Academy of Medicine, November 27, 1873.*

This comparatively insignificant ailment, the effects of which have never been known to destroy life, is one of the most troublesome little evils the physician is called upon to rectify. It is one of the numerous ills that result from fashionable folly, pride, and ignorance.

It exists only among civilized nations, and especially in the class of people whose pride impels them to undergo much physical discomfort, for the sake of exhibiting their feet neatly and fashionably clad.

The profound wisdom, and the delicately refined tastes of the highstocracy, or *bon ton*, would not (if they had the power,) allow people to be born with the tendency to large soles, and however small soles may be by nature made, fashion's imperious mandates direct them to be placed in still narrower limits than nature designed. The foot thus tightly encased, must be raised at the calcaneal extremity from two to four inches above the phalangial end, so as to increase the delightful pressure of the shoe or boot.

It is fortunate for the foolish followers of the Goddess of Fashion, that this compression is ordered at the low extremity of the body, for were it required at the upper end, the bills of mortality would be notably increased when we remember that invariable law or principle of pathology, that the weaker organs suffer more than the stronger, from the causes of disease.

Pride and vanity are evidences of mental weakness. The poet has truly said,

> "What the *weak mind* with *strongest* bias rules,
> Is *pride*, the never-failing vice of fools."

There is quite an obtuseness in the perception of truth in these fashionable simpletons. Did ever a physician know, among his lady acquaintances, one that would admit that she drew her corset too closely, or that her gaiters were too small?

Or did ever a vain-glorious fop, though he spend half an hour to get his feet into his twenty dollar moroccos, admit that his boots were tight fitting?

Hardly any one whose toes blossom with corns, will concede that they came from close fitting boots or shoes. Truth with them is swallowed up in foolishness.

I will excuse myself for making some comments on the use and abuse of foot coverings, since I believe that the subject is, by many, not fully understood; not from any special abstruseness in the matter, but simply because it has not received that amount of thought and attention that it deserves. In the first place, I will state, that nature, strictly followed, requires no covering for the feet that will interfere with their growth or freedom of movement. A *rigid adherence* to nature's wants, would allow the use of no covering, except what is required to protect the feet from the inclemencies of the weather. The customs of modern society, however, demand, as a matter of decorum, that the feet be clad at all seasons.

If, however, the precept be complied with, that the freedom of movement be unrestrained, and the feet costume conform to season and circumstances, being light and thin in the warmer, and thicker and heavier in the colder seasons, and in addition to these rules of freedom, and variable thickness of foot gear, and other known hygienic laws in regard to the case of the feet be observed, such ills as corns, bunions, and toe ulcers, would be great rarities. In the practice of society the foregoing conditions seldom exist. The feet, at all seasons, are *firmly compressed* by boots or shoes, and during the warm months, the boots and shoes worn by the male sex are of nearly the same quality as that worn by them during the colder months. The effect of wearing these heavy and close fitting boots and shoes during the warm weather, is, to enfeeble the growth of the skin, the epidermis and nails being imperfectly formed.

The compressed condition of the feet, the excessive heat and moisture from the retention of perspiration, the absence of the healthy stimulation of pure air, and the combined debilitating influences that result in a thin weak skin, small imperfect nails, little, sparsely scattered, abreviated hairs, and extremely thin epidermis, that ornament the nether extremeties of the city fop. Compare *his* feet with *those* of some brawny *backwood's man* that never wore a boot or shoe. Here is a distinction *with* a difference. Look at the toes on the fop's withered feet. Between the toes where the pretty narrow-pointed morocco boots produced a lively pressure, the epidermis is so light and thin that it can just be seen as a tissue paper-like covering. See the flesh between the toes crowded up by the pressure, above the level of the nails.

Is it any surprise that the sides and end, eʌen of the nail, (when the nail is kept closely trimmed,) is *pressed through* the delicate epidermis, producing irritation, inflammation, ulceration, suppuration, and, I had almost said, damnation, of the tissues beneath? This is, in probably most cases, the simple pathology of ingrowing toe nail. The nail does not grow into the flesh or toe, but the epidermis being thin and softened by continued moisture, the nail is *pressed through* it into the flesh. Then an opening being made, the presence of the nail keeps up the irritation and inflammation with all its results, as above mentioned, and of enlargement, (if the irritation is long continued,) of the side of the toe. The flesh surronnding the seat of the ulcer becomes hypertrophed, and which hypertrophy is sometimes an obstacle in treatment. Before detailing the treatment of ingrowing toe nail, I will present a few brief rules for the hygienic care of the feet:

1. The stocking or sock should be of ample size, and free from seams and irregularities.

2. The boot, shoe or gaiter, or whatever is worn over the sock, should likewise be ample in every part.

The only exception to this is, in cases in which (the feet being very tender,) friction provokes the formation of corns. In these cases, the boot or shoe should fit sufficiently close around the instep, and around the heel and instep, to lessen, partially, the motion of the boot or shoe on the foot.

The manifest objection to even this degree of tightness in the fit of the boot, is the impediment to freedom of movement, and the impossibility of free ventilation. The boot, as an article of wear, is a cursed nuisance. If it is made to prevent undue friction on the anterior part of the foot, the closeness around the instep is unpleasant and hurtful, and the odor arising from the feet on withdrawing the boot at night is noisome and hideous.

3. Practice daily, evening ablution.

With many persons the secretions from the enfeebled skin become foul and unhealthy. Ablution, frequent and thorough, is with them an essential requirement.

4. As far as practicable, supply the feet with an abundance of fresh air. Ventilation is demanded as much for the feet as for any other part of the body, and yet they receive less. The slipper should be substituted for the boot or shoe, especially during the evening hours, and at all times when permissible.

5. *Allow the nails to grow over the ends of the toes as a protection.* Never cut the nails close, and trim them straight across, leaving the corners at either side. The observance of the foregoing common-sense rules will almost invariably ensure freedom from corns, bunions, and toe ulcers, that now afflict thousands of Adam's race.

### *TREATMENT OF INGROWING TOE NAIL.*

The pathology being understood, the treatment will be readily indicated. We have shown, 1. That the epidermis is unnaturally thin, and of feeble resistance; 2. That the nail is pushed through the thin epidermis; 3. An ulcer is produced by the irritation of the nail, provoking inflammation with many or all of the conditions resulting from it; 4. If the irritation and inflammation had been of long continuance, an hypertrophed state of the skin and flesh in the vicinity of the ulcer arises. Those conditions being understood, the indications for treatment are simple and plain. They are,

1. Remove all pressure from the toe. Pressure is the first and chief cause of the trouble.

2. The nail should be permitted to grow without trimming, except what is done by the surgeon.

3. Remove the pressure of the edge of the nail upon the ulcer.

4. Heal the ulcer by giving it just the same treatment that you would give an ulcer elsewhere having the same pathological conditions.

5. After the ulcer is healed, promote the formation of healthy skin, especially the development of a thick, healthy, resisting epidermis.

If the surgeon retains his common sense, and use judiciously the ordinary therapeutic principles for the treatment of ulcers, there can be no doubt of success. The only additional condition being the pressure of the nail acting as a foreign body, pressing upon the ulcerated surface and keeping up the irritation. The contact of the nail may be obviated by pressing under the free point or border of the nail next to and upon the ulcer, a small bit of lint, or a small piece of compressed sponge, or if the anterior free edge projects forward sufficiently, this part may be elevated, by passing under it the whole width of the nail, a piece of sponge, cork, lint, or other material, and retained in place by adhesive strips passed lengthwise over the end of the toe.

Various ingenious contrivances have been suggested and used for separating the nail from the ulcerated surface. Those means first mentioned above are simple, easily applied, and as effectual as any that have been reccommended, and are, I think, to be preferred.

The removal of the *free unattached* border of the nail next the ulcer with the scissors, may be had recourse to if it is found difficult to separate the nail from the ulcer by the above method.

When the ulcer is in a state of inflammatory exacerbation, the least touch to the ulcer, or the least motion of the toe causes acute pain. No attempt should be made to separate the nail and ulcer until this is relieved.

Rest and the warm water dresssing will remove this condition, or the surgeon may apply freely to all parts of the ulcer the solid nitrate of silver, and follow the application with an emolient poultice. Caustic applications, usually, are not advisable during the height of the pain and inflammation. The application is for a short time intensely painful, and sometimes the inflammatory action is increased. The use of some form of caustic or stimulating application to the surface of the ulcer, used at intervals of three or four days for the stronger caustics, or daily for the milder ones, will aid much the progress of cure. Of the strong caustics the actual cautory has been used by the French. The American patient would not willingly submit to so powerful an agency for so trivial a malady. Nitrate of silver is more generally used than any other, and its action can be regulated in severity or mildness by using it of various dilutions, so that it can be made to cover the whole range of caustic applications.

If the ulcer does not demand a strong caustic, I should reccommend, from a limited experience, a trial of a saturated solution of alum in hot water, applied as hot as can be borne. This was first reccommended by Mr. Ure.—(See *London Lancet,* June, 1854, p. 530.) The only other instance recorded of its use in these cases that I can find, is by Dr. J. G. Poiter, of New London, Conn.—See *American Journal of Science,* for January, 1860, p. 126.

The application is not painful. It seemed to act well in the following case. Mrs. T., during the winter of 1870 and 1871, requested my advice. The great toe of the right foot presented on the outer side an ulcer, in a state of acute inflammation and swelling and pain. The parts were so sensitive that she would allow of no examination further than the sight would admit. It was merely an ingrowing toe nail with the ulcer and adjoining tissues in a state of acute inflammation, occurring in a sensitive nervous female. I directed the preparation of a saturated solution of alum in boiling water, and that the toe be enveloped in cloths wet with this solution as hot as could be borne. This application was kept up for forty-eight hours, the swelling and pain rapidly subsided. I then directed the application daily of the Tr. Ferri Chloridi U. S. D. The ulcer cicatrized rapidly, a firm epidermis was developed, the nail allowed to grow over the end of the toe, and the lady has since had no further trouble. The recommendation that I have invariably made to those who ask advice in regard to commencing trouble of ingrowing toe nail, is to avoid cutting the nail, remove pressure and apply freely once daily the Tr. Ferri Chloridi to the tender points at the side of the toe, introducing the fluid under the border of the nail as much as possible. This application causes momentary smarting and pain, which is followed by relief from tenderness. The epidermis becomes thick and resisting the toe nail under slight pressure, not wounding or breaking through it. The cases in which I have advised its use were relieved and the trouble averted.

Other preparations of iron have heen recommended, and I believe are equally beneficial. Dr. L. P. Babb, *Boston Medical and Surgical Journal,* July 9, 1868, p. 362, states that he used with invariaole success, a *saturated* solution of persulphate of iron. He introduces under the edge of the nail a small piece of cotton wool wet in the solution.

Dr. M. Billon, of the English army, see *Braithwait's Ret. of Medicine and Surgery,* January, 1864, p. 110, seeing the report of a case successfully treated by Dr. Caillett, by use of sesqui chloride of iron, used this agent. He inserts the *dry* sesqui chloride of iron between the nail and the flesh. He says the cure is "*prompt and painless.*"

"On the following day the exhuberant flesh is found to have acquired the hardness of wood, suppuration speedily ceases, and a cure follows after two or three applications."

The excision of a portion of the nail on the diseased side of the toe with a corresponding portion of the matrix is advocated and even practiced by some surgeons in a respectable attitude of authority, but on what grounds it is difficult to conceive. If the matrix and nail were *diseased,* there might be a small show of reason for this operation, provided the diseased condition was not ameniable to constitutional or local means. The removal of a *healthy* nail is supreme nonsense. If the same bad treatment of the feet and nails is afterward adopted by the patient that produced the trouble primarily, the disease will recur, unless the *whole nail* be removed and the *nail forming surface completely destroyed.* There are cases in which a removal of a portion of the tissues by the side of the toe is advisable. I refer to those cases in which there is an exhuberant hypertrophy of the soft parts which exhuberance has resulted from a long continued irritation and inflammation, and which persists in spite of local and constitutional means. This operation is advised and described by Dr. B. E. Cotting, *Boston Medical and Surgical Journal*, January, 1873, as follows:

One operation consists in removing with the knife, by a single stroke, all the diseased parts, together with quite a large piece of sound flesh, skin deep, from the side of the toe, sometimes making an open wound of, say, nearly an inch long by three-fourths of an inch wide."

This removes all hypertrophed tissue and the contraction of the subsequent cicatrix draws the flesh away from the nail.

The operation of removing the *healthy nail* is wholly unjustifiable unless it can be shown to be justifiable for a surgeon at a clinic, short of cases in operative surgery, finds among the out door patients, a poor fellow with an ingrowing toe nail that a kind Providence has sent to be tortured for the questionable benefit of the class.

I know that it is stated by some authorities that the nail is too much curved or grows out too far at the sides in some of these cases, but those who *have investigated the truth of the matter by examination of toes with and without* this malady, state that these peculiarities exist to so great an extent among those who never suffer from ingrowing toe nails as among those who do. See *American Journal of Science*, vol. 9, p. 166, *et seq.*

Since writing the foregoing, I have found in the *Scientific American*, January 28, 1871, p. 65, a short article on "ingrowing toe nail, quoted from Bostwick's Medical and Surgical Journal, in which the use of Perchloride of iron is highly recommended. Its use is spoken of either in the solid form or in solution.

The effect of the iron upon the skin is to thicken and harden it, especially the epidermis, so that it can resist the pressure of the nail. I believe that it is this property that it seems to possess in common with alum, only in a greater degree that rendered it chiefly valuable. I know that in those cases in which the skin under and next the nail being softened and weakened by the habits I have referred to, and the part is tender and sensitive, but no ulcer as yet formed, that the use of the Tr. Ferri Chloride will harden and thicken the epidermis and prevent the formation of toe ulcer. I hope the profession will make a fair trial of these agents, alum and iron, in its various forms mentioned, and I believe that the favorable effects I have mentioned will be realized. These agents are not only curative but preventive. They act promptly and surely. I believe that every one who use them will become possessed of that full confidence in these powers that the writer has.

